# Review of Literature: Genes Related to Postaxial Polydactyly

**DOI:** 10.3389/fped.2015.00008

**Published:** 2015-02-11

**Authors:** Prashant Kumar Verma, Ashraf A. El-Harouni

**Affiliations:** ^1^Department of Genetic Medicine, Faculty of Medicine, King Abdulaziz University, Jeddah, Saudi Arabia; ^2^Department of Clinical Genetics, National Research Center, Cairo, Egypt

**Keywords:** postaxial polydactyly, molecular approach, hedgehog protein, sonic hedgehog, signal pathways, gene, cilia, investigation, approach

## Abstract

**Background:** Postaxial polydactyly (PAP) is one of the commonest congenital malformations and usually is associated to several syndromes. There is no primary investigational strategy for PAP cases with single gene disorder in literature. PAP cases with single gene disorder can be classified according to common pathways and molecular basis. Molecular classification may help in diagnostic approach.

**Materials and Methods:** All single gene disorders associated with PAP reported on PubMed and OMIM are analyzed and classified according to molecular basis.

**Results:** Majority of genes related to cilia structure and functions are associated with PAP, so we classified them as ciliopathies and non-ciliopathies groups. Genes related to Shh–Gli3 pathway was the commonest group in non-ciliopathies.

**Conclusion:** Genes related to cilia are most commonly related to PAP due to their indirect relationship to Shh–Gli3 signaling pathway. Initially, PAP may be the only clinical finding with ciliopathies so those cases need follow up. Proper diagnosis is helpful for management and genetic counseling. Molecular approach may help to define pleiotropy.

## Introduction

**Figure 1:**
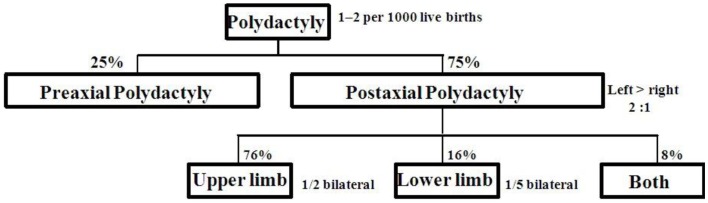
**Limb involvement in postaxial polydactyly**.

Postaxial polydactyly (PAP) is defined as an extra digit or a part of digit on the ulnar or fibular side of hand or foot. A small projection of tissue or scar mark just below the proximal interphalangeal crease can also be the only clinical finding. Prevalence of PAP is 1–2/1000 live births with some difference in ethnic groups (1, 2). PAP is more common (75%) than preaxial polydactyly (25%). About 8% of cases with bilateral PAP in upper and lower limbs are frequently associated with multiple congenital anomalies. Distribution of PAP is shown in Figure 1 (3). PAP is clinically classified into type A with fully developed extra digit and type B with incompletely developed digit (4). Type B PAP is commonly associated with isolated familial PAP (5).

Many congenital malformations associated to PAP are reported in literature (6–12). There are no reported guidelines or protocols to investigate these malformation syndromes.

**Figure 2:**
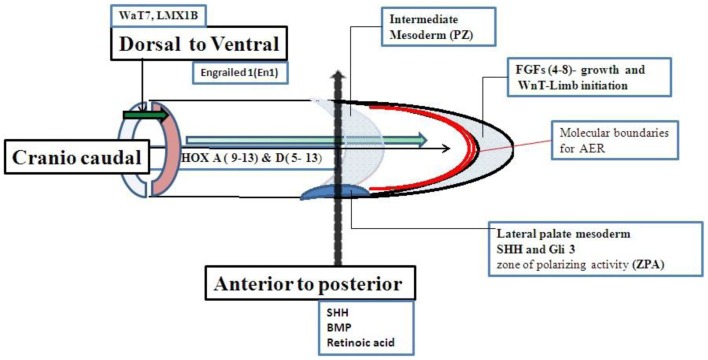
**Genes related to limb growth**.

Limb growth is controlled by a set of genes. The limb buds grow in three directions. The axis of growth is proximal to distal, dorsal to ventral, and anterior to posterior (first to fifth digit). Although a set of genes for limb growth are interacted to each other but the genes more specifically related to anterior to posterior axis shown in Figure 2 (13–27) are strongly related to molecular basis of PAP. Shh–Gli pathway is the well known pathway related to anterior to posterior growth pattern. Single gene disorders associated with PAP may be directly or indirectly related to Shh–Gli pathway. Classification of all reported single gene disorders associated with PAP on the basis of molecular association may help in making a common approach for investigation and genetic counseling of PAP.

## Materials and Methods

By using Mesh term “postaxial AND polydactyly” in searching PubMed and OMIM, we got total 667 entries. We included genetically well known syndromes with PAP and classified on the basis of common pathways and molecular association. We did not include single case reports and cases without molecular study. We also did not describe all phenotypic series of particular syndrome if genes are functionally related as Meckel, Bardet–Biedl syndrome (BBS), etc. As for clinical and molecular references for syndromes with PAP, we used NCBI resources like OMIM number, Gene ID, and relevant references related to gene function. We tried to define interactions between these genes for understanding the molecular mechanism how to PAP as related phenotype with particular gene.

## Results

Total of 36 genetically well known syndromes and entries were identified in which 16 (44%) related to ciliopathies group (Table 1) and 20 (56%) were unrelated groups and we classified them as non-ciliopathies group (Table 2). Most of the genes related to formation and development of embryo. Shh–Gli3 signaling pathway was the commonest pathway involved with PAP. PAP is more frequently associated with ciliopathy.

**Table 1:** **Ciliopathies (genes related to cilia biogenesis, structure, and functions)**.

S. No.	Genetic disorder	OMIM No.	MOI^a^	Gene	Gene ID	Gene function	Reference
1.	Acrocallosal syndrome	200990	AR	*KIF7*	374654	Negative and positive regulator of Shh pathway	(28)
2.	Asphyxiating thoracic dysplasia (Jeune syndrome) type II	611263	AR	*IFT80*	57560	Cilia motility and sensation	(29)
3.	Bardet–Biedl syndrome (I–XV)	209900	AR	*Heterogeneous*	–	Cilia biogenesis and function	(30)
4.	Cranioectodermal dysplasia 3	614099	AR	*ITF43*	614068	Cilia transport	(31)
5.	Cone–rod dystrophy 16	614500	AR	*C8ORF37*	157657	Cilia function	(32)
6.	Ellis-van Creveld (chondroectodermal dysplasia)	225500	AR	*EVC*	2121	Positive mediator of Shh	(33, 34)
				*EVC2*	
7.	Hydrolethalus 1 and 2	236680	AR	*HYLS1 and KIF 7*	219844	Core centriolar protein	(35)
8.	Joubert 20	614970	AR	*TMEM231*	79583	Diffusion barrier between the cilia and plasma membrane	(36)
9.	Joubert 14	614424	AR	*TMEM237*	65062	Ciliogenesis	(37)
10.	Kaufman–McKusick syndrome	236700	AR	*MKKS or BBS6*	8195	Ciliogenesis (mediator of BBSome complex assembly)	(38, 39)
11.	Meckel syndrome (1–10 types)	249000	AR	*MKS 1 to MKS 10 except MKS4 by CEP290*		Ciliogenesis	(40)
12.	Oral–facial–digital syndrome I	311200	XD	*OFD 1*	8481	Component of the centrioles	(41)
13.	Oral–facial–digital syndrome IV	258860	AR	*TCTN3*	26123	Ciliogenesis, Hedgehog signal transduction	(42)
14.	Short rib-polydactyl syndrome type II A	263520	AR	*NEK1*	4750	Involved in cell cycle cilium assembly	(43)
15.	Short rib-polydactyl syndrome type IIB and III	615087	AR	*DYNC2H1*	79659	Functions in cilia biogenesis intraflagellar retrograde transport	(44)
		263510	
16.	Weyers acrofacial dysostosis	193530	AR	*EVC*	2121	Positive mediator of Shh	(45)

*^a^Mode of inheritance (MOI) of most ciliopathies is autosomal recessive*.

**Table 2:** **Non-ciliopathies (genes not related to cilia biogenesis, structure, and functions)**.

S. No.	Genetic disorder	OMIM No.	MOI	Gene	Gene ID	Gene function	Reference
1.	Apert syndrome	101200	AD	*FGFR2*	2263	Embryonic patterning, limb bud development, etc.	(46)
2.	C syndrome	211750	AR	*CD96*	10225	Adhesive interactions of activated T and NK cells	(47)
3.	Carpenter syndrome 1/2	201000, 614976	AR	*RAB23*	51715, 1954	Silence the Shh pathway in dorsal neural cells	(48)
				*MEGF8*		Unknown	
4.	Chondrodysplasia punctata, X-linked dominant	302960	XLD	*EBP*	10682	Transport of cationic amphiphilics as integral protein of ER	(49)
5.	Chondrodysplasia, grebe type	200700	AR	*GDF5*	8200	Regulator of cell growth and differentiation in both embryonic and adult tissues	(50)
6.	Endocrine-cerebroosteodysplasia	612651	AR	*ICK*	22858	Intestinal epithelial cell proliferation and differentiation	(51)
7.	Fuhrmann syndrome	228930	AR	*WNT7A*	7476	During embryogenesis regulation of cell fate and patterning	(52, 53)
8.	Greig cephalopolysyndactyly, Pallister hall syndrome, PAP type A1 and type B	175700, 146510, 174200	AD	*GLI3*	2737	Mediators of Shh signaling	(54)
9.	Guttmacher syndrome	176305	AD	*HOXA13*	3209	DNA binding TF regulate during embryonic development like digit patterning	(55)
10.	IFAP syndrome with or without BRESHECK syndrome	308205	XR	*MBTPS2*	51360	Essential in development for activation of signal protein	(56, 57)
11.	Joubert syndrome 1	213300	AR	*INPP5E*	56623	Regulate Golgi-vesicular trafficking	(58)
12.	Loeys–Dietz syndrome, type 1A and 1B	609192, 610168	AD	*TGFBR1/TGFBR2*	7046, 7048	Signaling for transcription of genes related to cell proliferation	(59, 60)
13.	Megalencephaly-polymicrogyria-polydactyly hydrocephalus syndrome (MPPH)	603387	AD	*PIK3R2*	5296, 10000	Second messengers important in growth signaling pathways	(61)
				*AKT 3*		Regulators of cell signaling in response to insulin and growth factors	
14.	Otopalatodigital syndrome, type II (RARE)	304120	XD	*FLNA*	2316	Remodeling the cytoskeleton to effect changes in cell shape and migration	(62)
15.	Postaxial polydactyly (PAP) type A	–	AR	*ZNF141*	7700	Not known	(63)
16.	Syndactyly, type IV	186200	AD	*LMBR1*	64327	Cis-acting regulatory module for Shh	(64)
17.	Simpson–Golabi–Behmel syndrome, type 1	312870	XR	*GPC3*	2719	Cell division and growth regulation, inhibited soluble hedgehog activity	(65, 66)
18.	Schinzel–Giedion midface retraction syndrome	269150	AD	*SETBP1*	26040	Involved in DNA replication	(67)
19.	Smith–Lemli–Opitz syndrome	270400	AR	*DHCR7*	1717	Cholesterol biosynthesis and so indirectly for Shh signaling	(68)
20.	Ulnar–mammary syndrome	181450	AD	*TBX3*	6926	Anterior/posterior axis of the tetrapod forelimb	(69–71)

## Discussion

Postaxial polydactyly is one of the most common congenital malformations and a *key feature* for dysmorphic syndromes. Genetic syndromes related to cilia dysfunction are called ciliopathies, and the majority of this group is associated with PAP. Most of ciliopathies related genes work together as common unit and any defect in one component leads to dysfunction of overall cilia function, either directly or indirectly. This is the cause for overlapping clinical phenotypes of different ciliopathies. We were not discussing the complex genetics of human ciliopathies but focusing more on the molecular mechanism for PAP association with ciliopathies.

Genes associated with anterior to posterior patterning may be responsible for molecular etiology of PAP (Figure 2). Cilia should be involved with the genes associated with anterior to posterior patterning of the limb. The Shh–Gli3-activated Ptch transcription pathway is the most important pathway related to control anterior to posterior patterning and associated with PAP. Shh, Ptch 1, Smo, and Gli3 are the main genes in Shh–Gli3 pathway. Bone morphogenic protein (BMP) and retinoic acid are also needed for anterior to posterior patterning but their association with PAP is not reported in the literature.

Sonic hedgehog mutations are usually not reported with PAP in humans because most of these mutations are heterozygous. Haploinsufficiency of Shh gene does not affect the long range process of patterning (72).

Ptch 1 and Smo are the other intermediate genes in this pathway and both of them have an inhibitory function in Shh–Gli3 pathway. Mutations in these genes were not reported with PAP. Homozygous mutations in Ptch 1 and Smo are lethal during embryonic development and haploinsufficiency do not affect long range process of patterning (73, 74).

**Figure 3:**
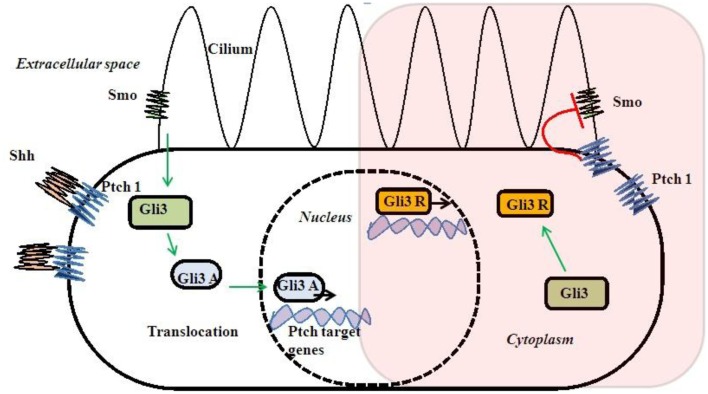
**Shh–Gli3 signaling and Ptch–Smo interaction and dual transcription**.

Gli3 gene is the most important gene in this pathway and mutations in this gene are reported with PAP. Gli3R is a repressor form without Shh signaling. Smo activated Gli3R to an activated Gli3A form after Shh–Ptch interaction due to loss of inhibitory effect of Ptch on Smo (Figure 3). Gli3 works as a dual function transcription factor. These two forms of Gli3R and Gli3A and their proportion of Gli3R/Gli3A forms directly are related to digit types and number (75, 76). Complete regulatory mechanism of the Gli3R/Gli3A ratio is still unclear. There is no exact genotype and phenotype correlation with Gli3 mutations due to complex interaction to other genes and bifunctional transcriptional switch (77, 78).

Sonic hedgehog pathway needs cilia for signaling (79, 80) (Figure 3). So, Shh–Gli3 signaling pathway is affected with most of the ciliopathies with PAP association and that may be due to altered Gli3R/Gli3A ratio. Cell lacking cilia or alteration of intraflagellar transport (IFT) cause changes in Gli3 processing and unable them to proceed Shh signaling (81, 82). Those single gene disorders associated with PAP directly or indirectly alter Gli3 signaling causing them to have some rational pleiotropy for PAP association.

**Figure 4:**
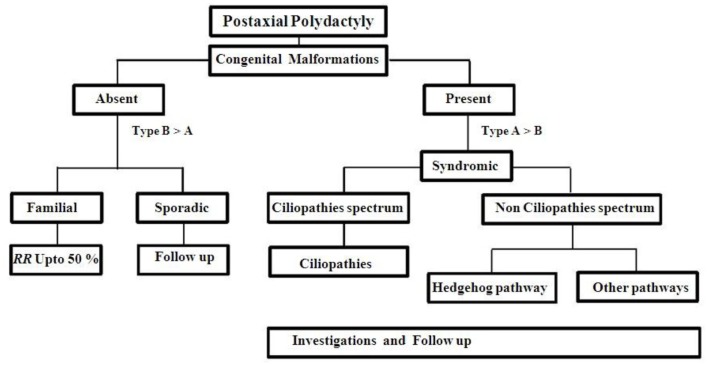
**Approach to a PAP case**.

Besides PAP, there were few cases reported overlapping in their clinical features with Gli3 and ciliopathies (83). For example, acrocallosal syndrome has some overlapping features with GCPS (Greig cephalopolysyndactyly syndrome). This may be due to KIF7 gene, which has negative or positive regulator mechanism in Shh pathway and needs molecular testing to confirm the diagnosis (84). PAP may be the only external malformation appreciated at birth, while other features may develop later in many ciliopathies. So, we made an investigation approach chart (Figure 4) during the first visit of any patient with PAP to the genetic clinic. Any patient with non-familial symmetrical PAP, even without congenital malformations, should be thoroughly investigated to rule out associated complications of ciliopathies (85) (Table 3). The recurrence risk for all ciliopathies is 25% per each pregnancy except OFD 1, which inherited as an X liked dominant trait.

**Table 3:** **Primary investigation protocol for a case of post axial polydactyly with malformations**.

Routine hemogram
Liver function test
Kidney function test
X ray of both hands with wrist
X ray feet
Chest X ray
Pure tone audiometry
Fundus examination
Ultrasound of abdomen
ECHO heart
MRI brain (*Mid line defect*+)
Karyotype (*Microarray preferable*)
FISH for 22q11.2
DNA banking

Single gene disorders other than ciliopathies associated with PAP were classified as non-ciliopathies. In this group, functionally related genes to Shh–Gli3 pathways are Gli3, LMBR1, and DHCR7. GDF5 and TGFBR1, 2 genes are belonging to TGF-β signaling pathways. While WNT7A and FGFR2 genes are belonging to Wnt and FGF signaling pathways. Although WNT7A and FGFR2 genes interact with Shh pathway during limb development, but the exact molecular mechanism for PAP is still unclear. Most of other genes in this group belongs to gene families, which were not yet included in a specific pathway.

We also tried to find out the type of pleiotropy for PAP association. Pleiotropy is defined as multiple distinct phenotypic variants caused by a single gene. Most of these genes with PAP association are related to embryonic patterning and development. Rational and mosaic are the two most common types of pleiotropy. Genes, which have a molecular mechanism for explaining particular trait, are called rational pleiotropy whereas those not having it are called mosaic pleiotropy (86). In our study, we found out rational pleiotropy for PAP association only with syndromes is related to Shh–Gli3 pathway. Other syndromes may be having mosaic pleiotropy for PAP association.

Recurrence risk for familial autosomal dominant (AD) PAP is up to 50% per each pregnancy with variable expressivity. Non-familial case should be kept in follow up (Figure 4). Cytogenetic studies should be done for multiple congenital anomalies associated with PAP without specific dysmorphology. Chromosomes abnormalities in 2, 3, 4, 7, 13, 14, and 18 were reported with PAP (87–97). Single gene testing is not acceptable to most of ciliopathies disorders because of genetic heterogeneity, oligogenic inheritance, and age dependent penetrance. So, initially most of the cases are classified upon the clinical basis but further more investigations are necessary for proper diagnosis and genetic counseling.

## Conclusion

Genes related to anterior to posterior patterning are responsible for PAP. Dysregulation or mutations of the Gli3 gene was associated with PAP. Genes related to cilia are most commonly related to PAP due to their indirect relationship to Shh–Gli3 signaling pathway. Initially, PAP may be the only clinical findings with ciliopathies so these cases need continuous follow up.

## Conflict of Interest Statement

The authors declare that the research was conducted in the absence of any commercial or financial relationships that could be construed as a potential conflict of interest.

## Abbreviations

AD, autosomal dominant; AR, autosomal recessive; BMP, bone morphogenetic protein; DHCR7, 7-dehydrocholesterol reductase; FGFR2, fibroblast growth factor receptor 2; GDF5, growth/differentiation factor 5; GLI3, GLI-Kruppel family member 3; GLIR and GLI3A, GLI-Kruppel family member 3 activators and GLI-Kruppel family member 3 repressors; LMBR1, limb region 1, mouse, homolog of; MOI, mode of inheritance; OFD 1, orofaciodigital syndrome I; PAP, post axial polydactyly; PTCH 1, patched, *Drosophila*, homolog of, 1; Shh, sonic hedgehog; SMO, smoothened, *Drosophila*, homolog of; TGFBR1, 2, transforming growth factor, beta type 1,2 receptor; WNT7A, wingless-type MMTV integration site family, member 7A; XD, X-linked dominant; XR, X-linked recessive.
